# Growth hormone ameliorates the age-associated depletion of ovarian reserve and decline of oocyte quality via inhibiting the activation of Fos and Jun signaling

**DOI:** 10.18632/aging.202534

**Published:** 2021-02-17

**Authors:** Chuanming Liu, Shiyuan Li, Yifan Li, Jiao Tian, Xiaoling Sun, Tianran Song, Guijun Yan, Lijun Ding, Haixiang Sun

**Affiliations:** 1 Reproductive Medicine Center, The Affiliated Drum Tower Hospital of Nanjing University Medical School, Nanjing 210008, People’s Republic of China; 2 Center for Clinical Stem Cell Reasearch, The Affiliated Drum Tower Hospital of Nanjing University Medical School, Nanjing 210008, People’s Republic of China

**Keywords:** growth hormone, aging, oocyte quality, meiosis, apoptosis

## Abstract

Oocyte quality typically begins to decline with aging, which contributes to subfertility and infertility. However, there is still no effective treatment to restore the ovarian reserve and improve aged-oocyte quality. According to the present study, growth hormone (GH) secretion changes with maternal age in female mice. After intraperitoneal injection with GH (1 mg/kg body weight) every two days for two months, the 10-month-old mice showed a better ovarian reserve and oocyte quality than control mice. GH treatment decreased the occurrence rate of aneuploidy caused by spindle/chromosome defects. Additionally, the single oocyte transcriptome analysis indicated that GH decreased the expression of apoptosis-related genes in oocytes. It was also observed that GH treatment reduced the expression of γH2AX and apoptosis of aged oocytes via decreasing the activation of Fos and Jun. Collectively, our results indicate that GH treatment is an effective way to reverse the age-associated depletion of ovarian reserve and the decline of oocyte quality by decreasing apoptosis.

## INTRODUCTION

Female fecundability and pregnancy outcome are notably dependent on the conceiving age. Accompanied by the reduction in both ovarian storage and oocyte competency, women of advanced maternal age, particularly over 35 years, could consequently bear a greater risk of infertility, miscarriage, and chromosomal aneuploidy [1]. Telomere length, spindle assembly, mitochondrial dynamics, and chromosomal integrity are decisively related to oocyte competency and clinical outcome [2–4]. Though it is known that maternal age is aggressively associated with fertility, there is still no effective treatment to improve the ovarian reserve and oocyte quality in aged women [5].

Growth hormone (GH) is a single chain polypeptide and mainly generated by the anterior pituitary gland. It was first identified as a remarkable regulator of longitudinal growth and is also known for its multiple regulatory effects on the metabolism of lipids, glucose, and proteins and for modulating immune functions [6–8]. Its secretion rate exponentially decreases after physical maturation and is at a much lower level in elderly women than in young adults [9]. Inadequate GH secretion hinders muscle and skeletal development during adolescence, and can also result in insulin resistance or metabolic disorders in the liver and increase the cardiovascular risk in adults [10, 11]. Therapeutic use of GH enhances the function of many tissues in the elderly, such as skeletal muscle, the brain, and the heart [11–13].

GH plays important roles in sustaining normal reproductive abilities, and the expression of GH receptors (GHRs) has been detected in human oocytes and granulosa cells [14]. Through autocrine or paracrine signaling, GH exerts biological influences on folliculogenesis, oocyte maturation, and steroid synthesis [15, 16]. Besides, GH cooperates in the regulation of vascular endothelial growth factor A (VEGF-A), which is an essential mediator in ovarian angiogenesis [17]. Furthermore, reproductive deterioration is observed in maternal GH-deficient rat models, as indicated by irregular estrous cycles, reduced follicle number, and shrinking litter size [18]. Age-related decline in GH levels may be related to the insufficient reproductive potential in women of advanced maternal age. GH has already been applied as an adjuvant to enhance ovarian sensitivity to gonadotropin stimulation since the 1980s [19]. Nevertheless, its role in *in vitro* fertilization (IVF) remains controversial until today [20]. A meta-analysis by Li et al. showed that in poorly responding patients undergoing IVF, co-treatment with GH could significantly increase the number of MII oocytes and estradiol (E_2_) levels, shorten the stimulating duration before oocyte retrieval, and improve the rates of pregnancy and live birth [21]. Moreover, it has been proved that the expression levels of GHRs in granulosa cells evidently decrease in infertile patients over 30 years old, while GH treatment can effectively ameliorate the levels of GH, FSH, and LH receptors and ovarian storage and oocyte quality [22]. However, the increase of live birth rate in poor responders after GH treatment was not sufficient in a randomized controlled trial [23]. These outcome discrepancies can probably be attributed to different dosages, intervals, and timing of GH treatment, as well as the heterogeneity of patients. Therefore, more specific dosing regimens to achieve the optimal therapeutic effect are still under discussion [23, 24].

Due to the difficulty in observing follicles at different stages and the preciousness of MII oocytes derived from clinical patients undergoing IVF, there is a need to systematically verify the efficacy of GH treatment and investigate the underlying mechanisms in animal models. In the present study, it is shown that the decline in GH levels and the deterioration of oocyte quality result from maternal aging, and that both *in vivo* and *in vitro* treatment of GH restored reproductive function in mice. By single oocyte transcriptome analysis, we further determined that GH treatment can reduce apoptosis and DNA damage by decreasing the expressions of the *Fos* and *Jun* gene families.

## RESULTS

### Maternal aging causes a decrease of GH levels and deterioration of oocyte quality

**Figure 1:**
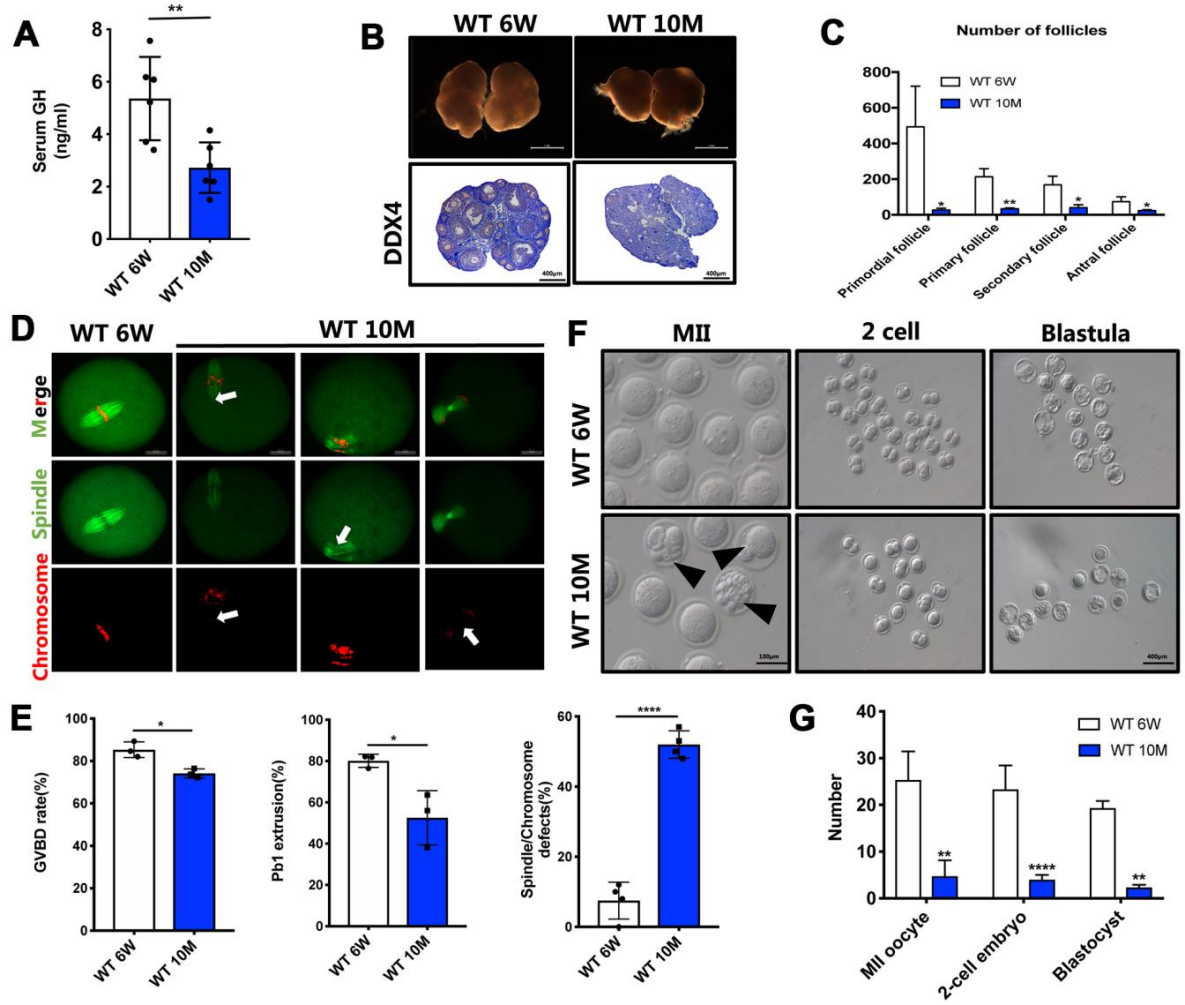
**The GH levels and oocyte quality were declined in aged mice.** (**A**) The GH levels in the peripheral blood were measured in young (n = 6) and aged (n = 6) mice. (**B**) Micrographs of young and aged WT mouse ovaries (Scale bar, 1 mm) and HE staining of these ovaries (Scale bar, 100μm, 500 μm). (**C**) Follicle counts in 6-week (n = 3) and 10-month (n = 3) old WT mice. (**D**) Chromosomes misalignment and spindle defects (arrowheads) in aged oocytes. The oocytes were stained with α-tubulin (green) and propidium iodide (PI) (red) respectively. Scale bar, 50 μm. (**E**) Left: The rate of GVBD and Pb1 extrusion were recorded after 4 h and 14 h of culture in M2 medium respectively. Right: Percentages of oocytes with spindle defects in young (n = 97) and aged mice (n = 104). (**F**) Representative images of MII oocytes collected from young (n = 76) and aged (n = 19) mice and IVF outcomes from these two groups. Black arrowheads point to abnormal oocytes. Scale bar, 100 μm, 400 μm. (**G**) Quantification of MII oocytes, 2-cell embryo and blastocyst from young and aged mice. Data are presented as mean ± SD. *P < 0.05, **P < 0.01, ****P < 0.0005.

We first investigated GH levels in peripheral blood during maternal aging, and the results showed a two-fold decrease of GH levels in 10-month-old mice (Figure 1A). Ovary tissues were then retrieved from both aged and young mice and assessed with HE staining. There were fewer and smaller oocytes in the aged ovaries (Figure 1B). On the basis of follicle counts, a prominent decrease in the number of follicles at all developmental stages was observed in aged mice compared with young ones (*P* < 0.05) (Figure 1C). In the meanwhile, the germinal vesicle breakdown (GVBD) rate (74.13%) and polar body extrusion (PBE) rate (52.57%) were severely impaired by maternal aging (Figure 1E; *P* < 0.05). Moreover, the frequencies of irregularly assembled spindles and misaligned chromosomes (52%) remarkably increased in aged oocytes compared with young oocytes (7.5%) (Figure 1D, 1E; *P* < 0.0005). Additionally, after MII oocytes from young and aged mice were fertilized *in vitro*, more abnormal and fragmented oocytes were observed in aged mice. Importantly, the potential of MII oocytes derived from aged mice to develop into the two-cell stage and further into blastula embryos was also significantly reduced (Figure 1F, 1G; *P* < 0.01). Collectively, oocyte quality and their potential to develop into surviving embryos could be gravely impacted by advanced maternal age, which is accompanied by a reduction of GH levels.

### Treatment with GH improves the quality of aged oocytes *in vitro* and *in vivo*

**Figure 2:**
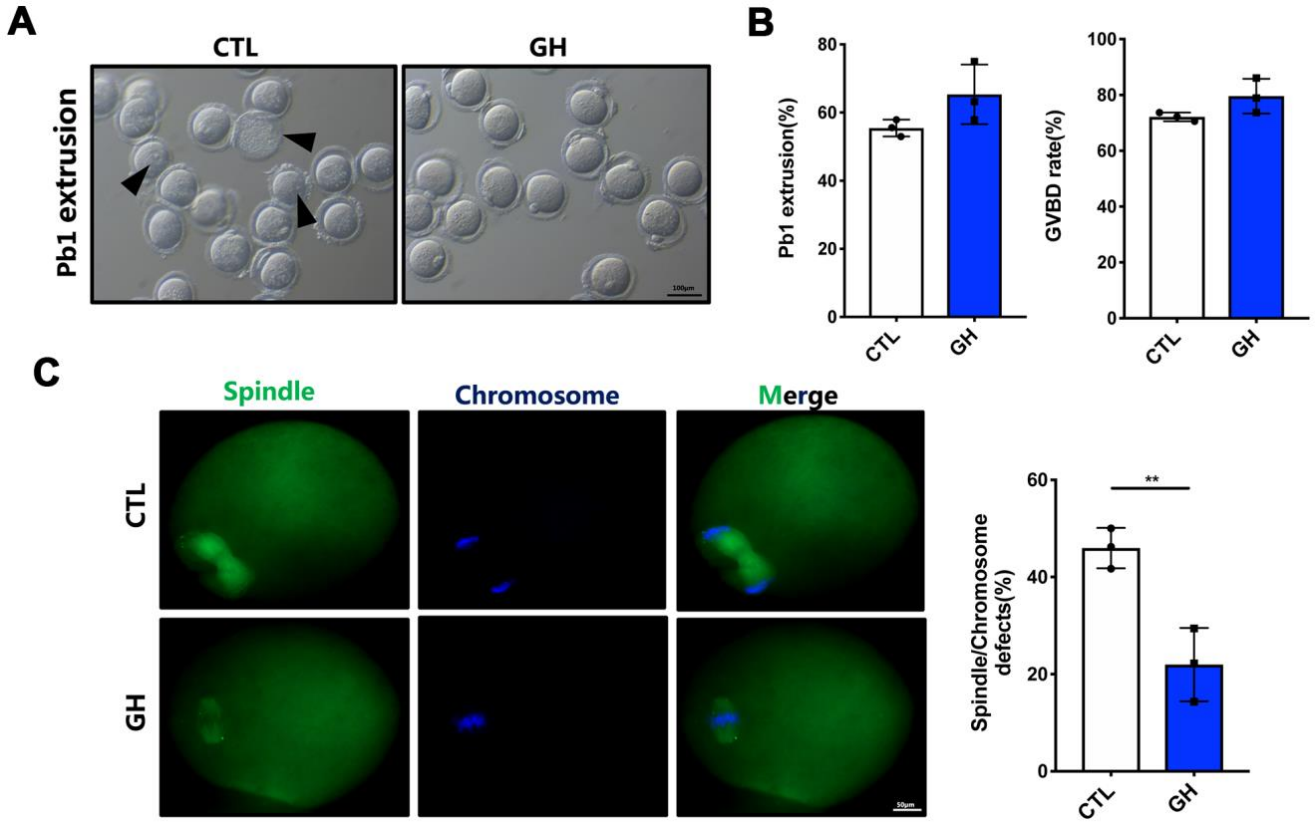
**Effects of GH treatment *in vitro* on the meiotic progress of aged oocytes.** (**A**) Representative images of oocytes after 14 hours cultured in M2 medium (n = 87) and GH treated medium (n = 88). Black arrowheads point to oocytes that fail to extrude a polar body or fail to survive. Scale bar, 100 μm. (**B**) The rate of GVBD and Pb1 extrusion in these two groups respectively. (**C**) Left: Spindle morphologies in control and GH group. Oocytes were stained with α-tubulin (green) and hoechst (blue). Scale bar, 50 μm. Right: Percentages of oocytes with spindle defects in control (n = 51) and GH group (n = 68). Data are presented as mean ± SD. **P < 0.01.

In order to assess the effects of GH on aged oocytes, oocytes were treated with GH (50 ng/mL) in maturation medium *in vitro*. We calculated the rates of GVBD and PBE after 4 and 14 h. There was an increasing tendency in the rates of GVBD (79.57%) and PBE (65.37%) compared with the control group (72.15% and 55.46%) (Figure 2A, 2B). Additionally, the incidence of disordered spindles with misaligned chromosomes (21.97%) was significantly lower in the GH group (Figure 2C; *P* < 0.01).

**Figure 3:**
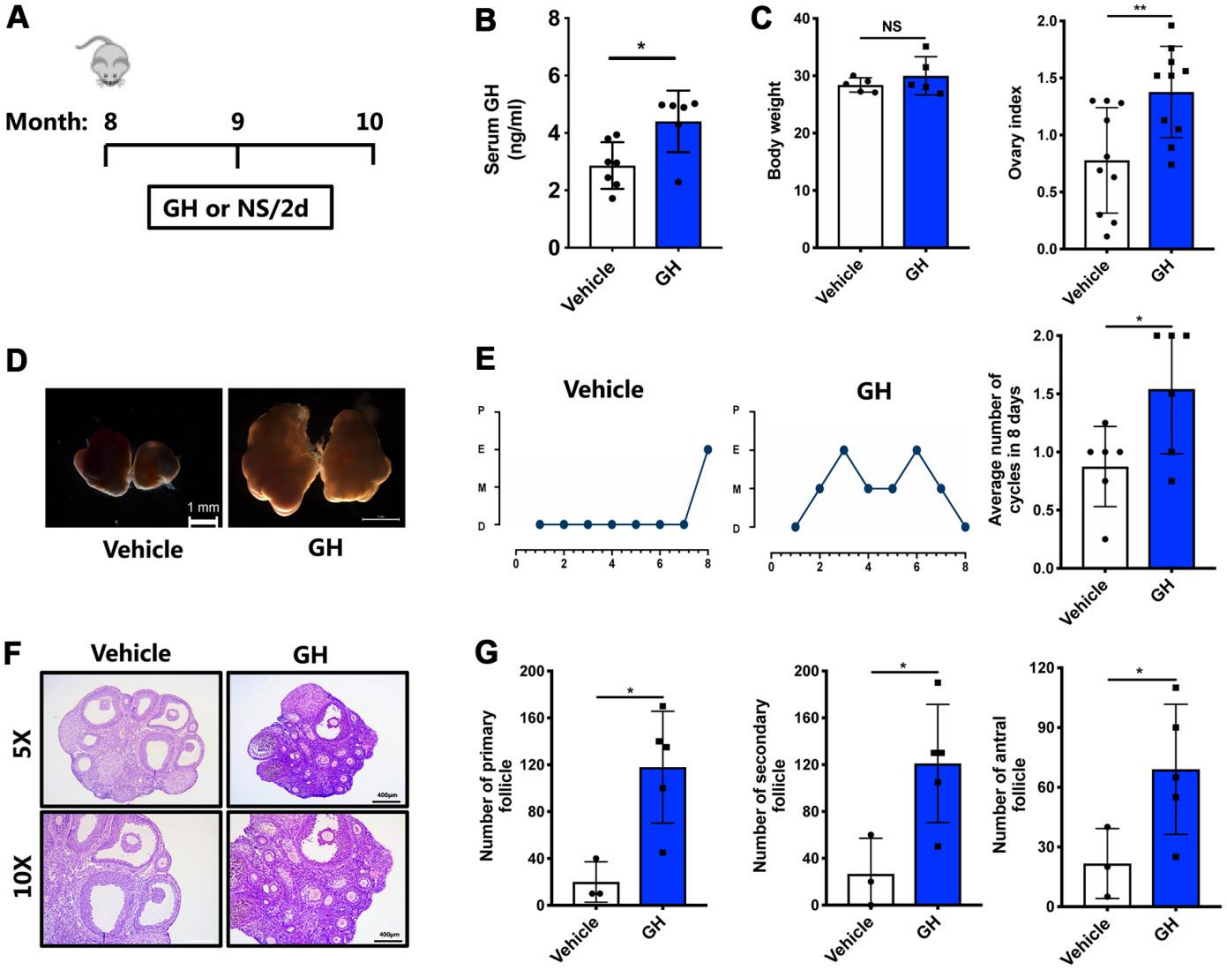
**Effects of GH administration *in vivo* on the ovarian reserve.** (**A**) Schematic illustration for the NS and GH-treated mice. (**B**) The GH levels in the peripheral blood was measured in GH (n = 6) and vehicle (n = 7) group. (**C**) The changes of body weight and ovary index after GH administration. NS, no significance. (**D**) Micrographs of NS-treated and GH-treated mouse ovaries. Scale bar, 1 mm. (**E**) Left: Estrous cycle in representative females. Right: Average numbers of cycles in 8 days in two groups. P, proestrus; E, estrus; M, metestrus; D, diestrus. (**F**) HE-stained of NS-treated and GH-treated mouse ovaries. Scale bar, 400 μm, 200 μm. (**G**) Follicle counts and the number of corpus luteum in NS-treated (n = 3) and GH-treated (n = 5) mice. Data are presented as mean ± SD. *P < 0.05, **P < 0.01.

To determine the curative effects of GH *in vivo*, 8-month-old mice were intraperitoneally injected with GH (1 mg/kg body weight) every two days (Figure 3A), while control mice were treated with normal saline (NS). After two months, GH levels were higher in the treatment group (4.404 ng/mL) than in the control group (Figure 3B; *P* < 0.05). The ovarian index was significantly higher in the GH group (Figure 3C; *P* < 0.01). As shown in Figure 3D, ovaries were enlarged after GH supplementation. Also, GH supplement seemed to enlarge the size of liver and heart., but made no difference. And it had no effect on the structure of liver, heart, uterus and oviduct (Supplementary Figure 1). With respect to the estrous cycle, mice in the GH group showed more estrus stages, and GH supplementation notably increased the average number of estrous cycles during eight days (Figure 3E; *P* < 0.05). Quantification of the hematoxylin and eosin (HE) staining results showed that the number of follicles at later growth stages, which deteriorated due to aging, was restored (Figure 3F, 3G; *P* < 0.05). Besides, the number of corpus luteum also increased after GH treatment (Figure 3G; *P* < 0.05).

**Figure 4:**
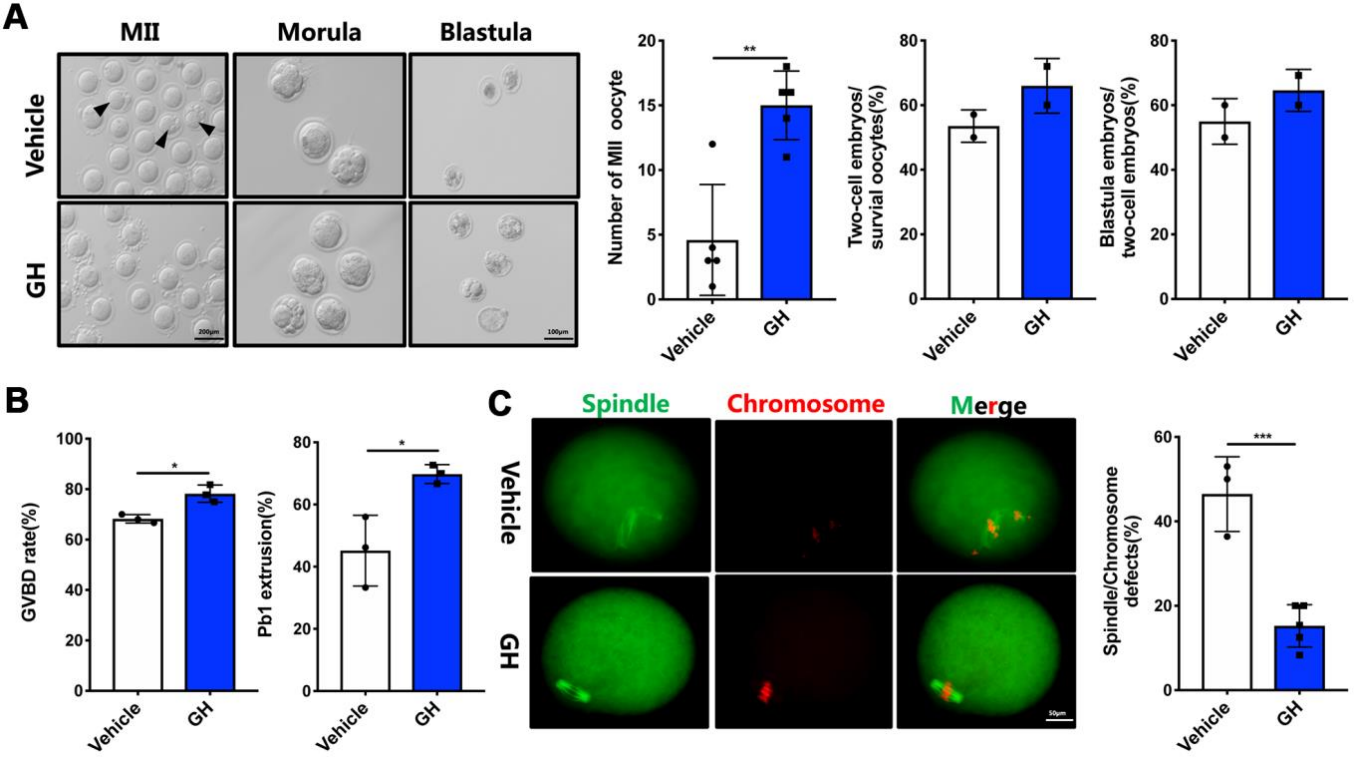
**Effects of GH treatment *in vivo* on the quality and meiotic progress of aged oocytes.** (**A**) Left: Representative images of MII oocytes, morula and blastocysts from NS-treated and GH-treated mice. Black arrows point to fragmented MII oocytes. Scale bar, 200 μm, 100 μm. Right: Number of MII oocytes and percentage of 2-cell embryos and blastocysts in NS-treated and GH-treated mice. (**B**) After cultured in M2 medium, the rate of GVBD and Pb1 extrusion were recorded. (**C**) Left: The MII oocytes from NS-treated (n = 37) and GH-treated (n = 42) mice were stained with α-tubulin (green) and propidium iodide (PI) (red). Scale bar, 50 μm. Right: Quantification of NS-treated and GH-treated oocytes with abnormal spindle/chromosomes. Data are presented as mean ± SD. *P < 0.05, **P < 0.01, ***P < 0.001.

In order to further test the potential effects of GH on oocyte quality *in vivo*, IVF experiments were performed using oocytes obtained from NS-treated and GH-treated mice. Apart from the morphology of oocytes, the number of MII oocytes (15 oocytes/mouse) and the ability to develop blastocysts (64.6%) were increased following treatment with GH (Figure 4A; *P* < 0.01). Furthermore, oocytes from mice treated with GH showed higher rates of GVBD (78.2%) and PBE (69.8%) than those of the control group (68.23% and 45.17%) (Figure 4B; *P* < 0.05). In addition, oocytes from the GH group presented a lower frequency of spindle and chromosome disorganization (15.25%) (Figure 4C; *P* < 0.001). Taken together, these results suggest that exposure to GH improves oocyte quality and prevents meiotic failure resulting from maternal aging.

### GH supplementation reduces oocyte apoptosis and the expression of γH2AX

**Figure 5:**
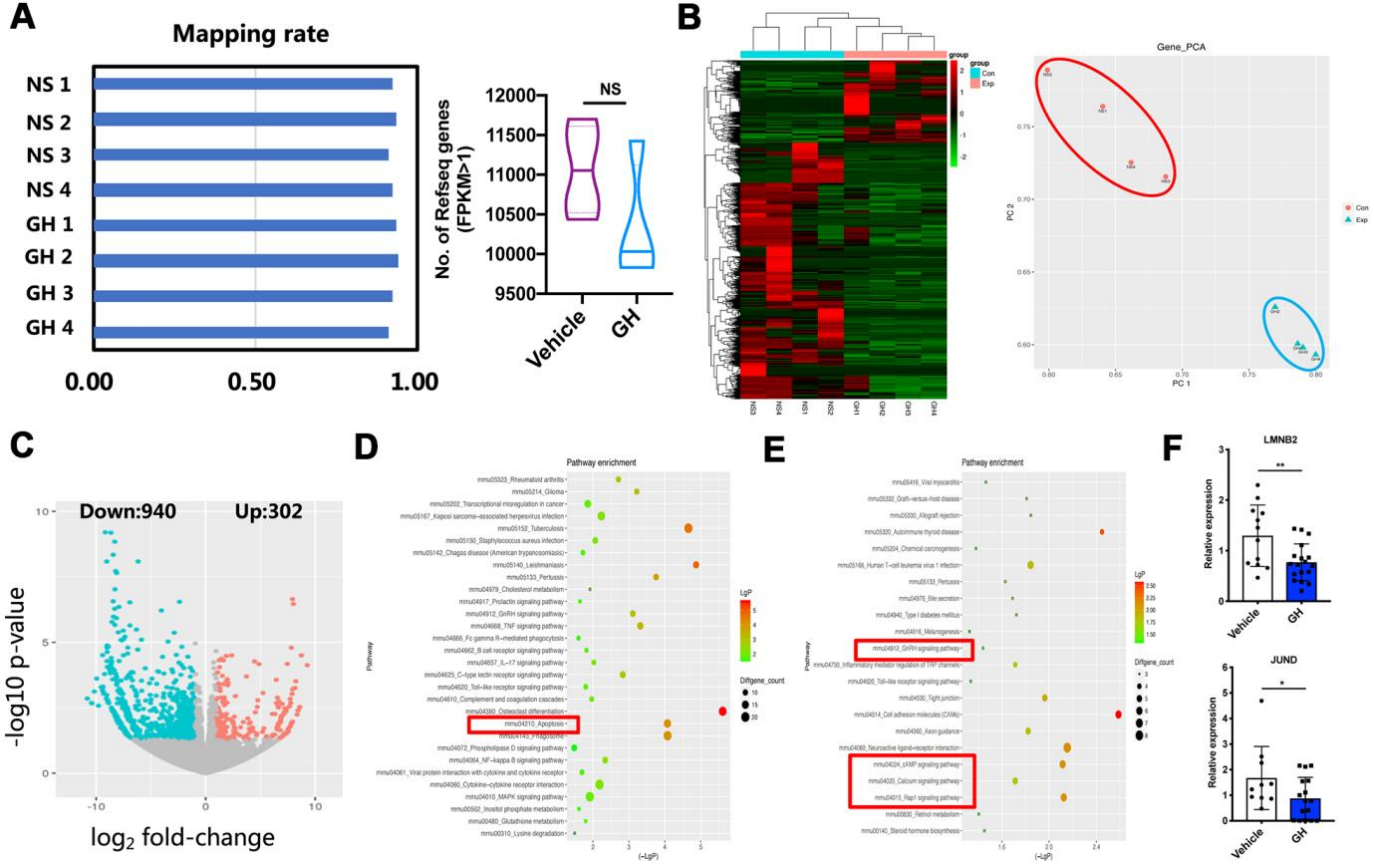
**ScRNA-seq of NS-treated and GH-treated MII oocytes.** (**A**) Left: Mapping rate of NS-treated oocytes (n = 4) and GH-treated (n = 4) oocytes. Right: The number of detected genes (FPKM > 1) in vehicle and GH group oocytes. NS, no significance. (**B**) Left: The gene expression heatmap showed the differentially expressed genes (DEGs) in these two groups. Right: Principal components analysis (PCA) of eight samples. (**C**) The volcano map showed the DEGs between NS-treated and GH-treated mice. (**D**) The top 30 KEGG pathways involved in the down-regulated genes. The red box encloses the apoptosis pathway. (**E**) The top 22 KEGG pathways involved in the up-regulated genes. The red box encloses the GnRH signaling, cAMP signaling, calcium signaling and Rap1 signaling pathway. (**F**) 2 DEGs were selected for QPCR validation (n ≥ 10). Data are presented as mean ± SD. *P < 0.05, **P < 0.01.

To investigate the mechanisms underlying the effects of GH treatment, single oocyte sequencing analysis was performed on four NS-treated and four GH-treated MII oocytes. As shown in Figure 5A, the mapping rate of each sample was above 90%. There was no significant difference in the number of expressed genes (FPKM > 1) between two groups, and principal component analysis verified that there were two clear groups, which corroborated the reliability of the data (Figure 5B). The transcriptome of oocytes from the GH group was remarkably different from that of oocytes from the NS group, as shown in the heatmap and the volcano plot. Of all differentially expressed genes, 940 genes were downregulated and 302 genes were upregulated after GH supplementation (Figure 5B, 5C Supplementary Table 1). The expressions of two selected differentially expressed genes were confirmed by single oocyte quantitative real-time PCR (qPCR) (Figure 5F; *P* < 0.05, *P* < 0.01). KEGG pathway analysis showed that the pathway associated with apoptosis, which is related to the survival and quality of oocytes, was significantly downregulated in the GH-treated group (Figure 5D). In contrast, among upregulated genes, the enriched KEGG pathways were associated with GnRH signaling, cAMP signaling, calcium signaling, and Rap1 signaling (Figure 5E).

**Figure 6:**
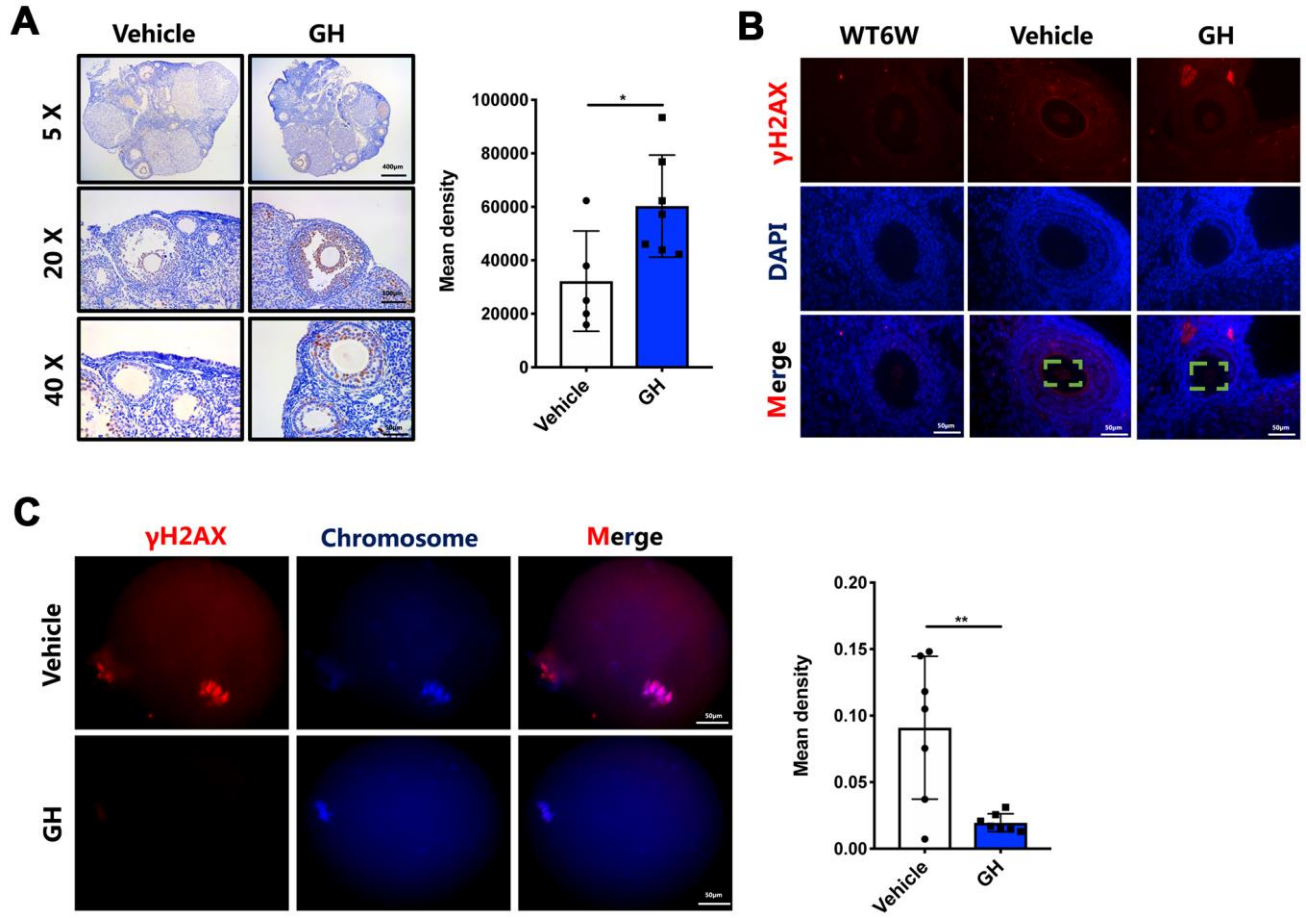
**GH treatment decreased oocyte apoptosis and DNA damage.** (**A**) Left: Immunostaining for Ki-67 in ovaries from the NS and GH groups. Brown represents positive staining. Scale bar, 400, 100, 50 μm. Right: The mean density levels of Ki-67 in ovarian. (**B**) The ovaries from NS-treated and GH-treated mice were stained with γH2AX (red) and DAPI (blue). Scale bar, 50 μm. (**C**) Left: The MII oocytes from NS-treated (n = 7) and GH-treated (n=7) mice were stained with γH2AX (red) and hoechst (blue). Scale bar, 50 μm. Right: Mean density levels of NS-treated and GH-treated oocytes. Data are presented as mean ± SD. *P < 0.05, **P < 0.01.

To confirm whether supplementation with GH could suppress apoptosis in aged oocytes, as indicated by the transcriptome analysis, Ki67 was stained to estimate the proliferation of granulosa cells. The proliferation index of the granulosa cells was higher in GH-treated ovaries compared with the NS-treated ovaries (Figure 6A; *P* < 0.05). In addition, γH2AX was highly expressed in NS-treated ovaries compared with young (6-week-old) mice and the GH group (Figure 6B). Immunofluorescence analysis revealed a decreased γH2AX in GH-treated oocytes (Figure 6C; *P* < 0.01). These results indicate that apoptosis of oocytes is decreased after treatment, and GH improves the proliferation of granulosa cells.

### GH treatment ameliorates quality of aged oocytes via regulating the expressions of the *Fos* and *Jun* gene families

**Figure 7:**
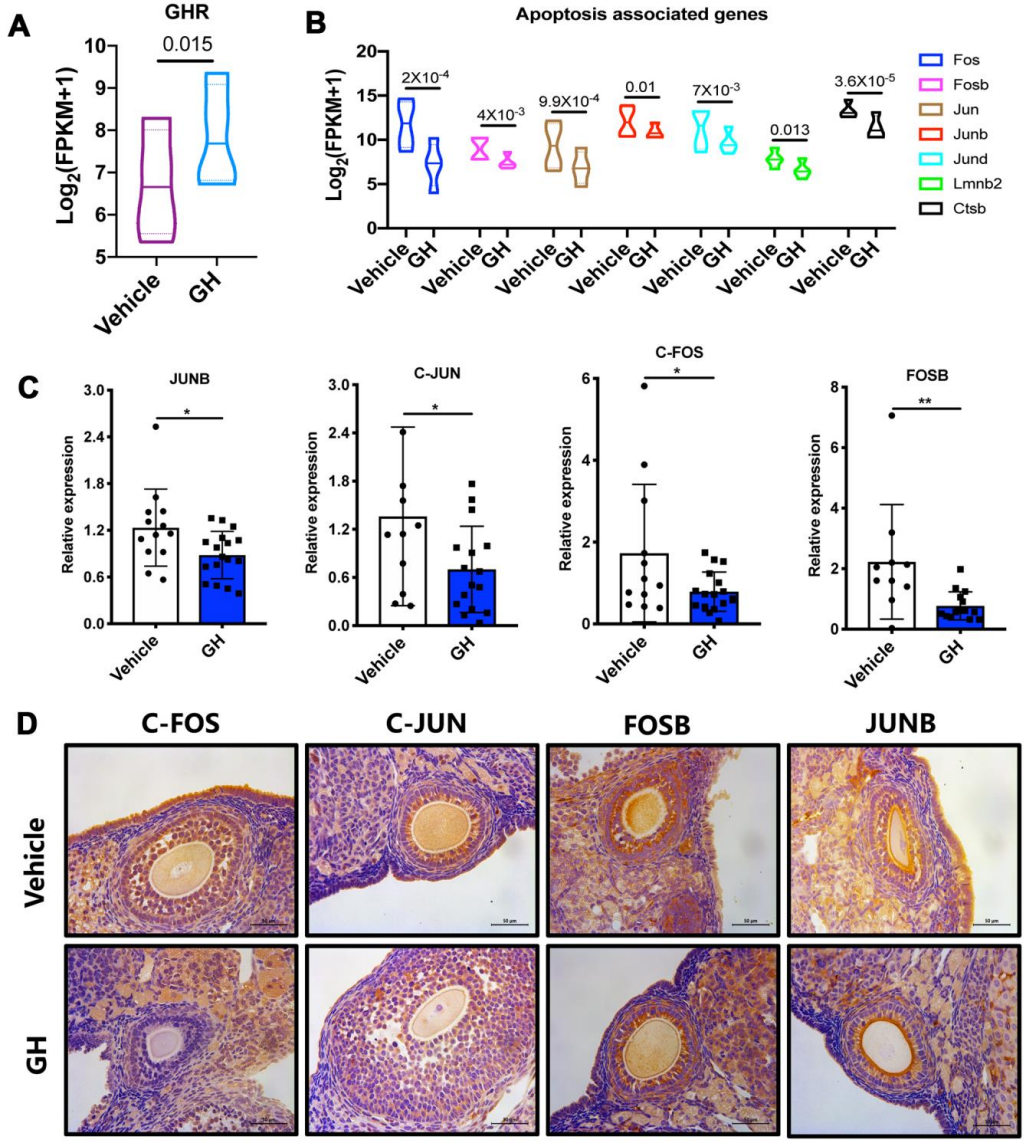
**Effect of GH treatment on the FOS/JUN pathway.** (**A**) The expression of GHR in GH-treated and NS-treated oocytes. (**B**) The expression of *Fos*, *Fosb*, *Jun*, *Junb*, *Jund*, *Lmnb2* and *Ctsb* in GH-treated and NS-treated oocytes according to the sequencing data. (**C**) Results of single oocyte QPCR in the two groups (n ≥ 10). (**D**) Immunostaining for *Fos*, *Jun*, *Fosb* and *Junb* in ovaries from the NS and GH groups. Brown represents positive staining. Data are presented as mean ± SD. *P < 0.05.

**Figure 8:**
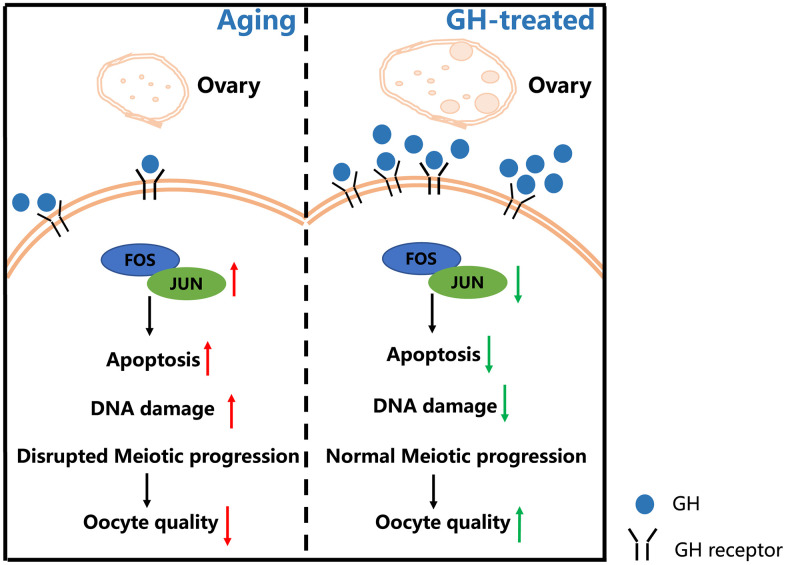
**Schematic diagram regarding how GH reverses the age-associated depletion of ovarian reserve and declining quality of oocytes.** In old mice, the ovarian reserve and the levels of GH are declined. The decreased GH enhances the expression levels of *Fos* and *Jun* family which increase the oocyte apoptosis and DNA damage. Administration of GH restores the depletion of ovarian reserve and induces the increment of GHRs. Additionally, GH supplementation exerts an influence on reversing apoptosis via lowering the expression of *Fos* and *Jun* which improves the oocyte quality.

To explore the mechanisms by which GH reduces oocyte apoptosis, GH signaling activity was analyzed. Higher expression levels of GHR were observed in the GH group (Figure 7A; *P* = 0.015). The *Fos* and *Jun* families play important roles in apoptotic cell death; the expression levels of genes such as *c-Fos*, *Fosb*, *c-Jun*, and *Junb* were greatly declined in oocytes under GH treatment (Figure 7B). The expressions of these genes in GH-treated and NS-treated oocytes were verified using single oocyte qPCR. The immunohistochemistry results further proved that GH could decrease the gene expression levels of the *Fos* and *Jun* families (Figure 7C, 7D). Taken together, GH treatment induced an increase in GHR levels in aged oocytes and inhibited apoptosis via lowering the expression of *Fos* and *Jun* (Figure 8).

## DISCUSSION

The changes in women’s role in society have brought a significant impact on the time of childbearing, increasing the rates of infertility and chromosomal aneuploidy [25]. An increasing number of studies are focusing on how to ameliorate the age-associated depletion of the ovarian reserve and improve the quality of oocytes. In the current study, the age-related decline in GH levels is shown to impair fertility, while after two months of GH treatment, the number of follicles and the developmental potential of oocytes are ameliorated. Moreover, spindle/chromosome defects and oocyte apoptosis were also reversed. This is the first study to systematically evaluate the effects of long-term GH treatment on the rejuvenation of aged ovaries.

It was recently documented that resveratrol, melatonin, and nicotinamide mononucleotide can enhance mitochondrial capacity in aged oocytes and restore oocyte quality [26–30]. GH is a natural anti-aging polypeptide that is frequently used to treat several aging-related diseases. It can also be beneficial to mitochondrial functions in aged mice, as it could increase ATP levels and improve the homogeneous distribution of mitochondria in oocytes [31]. *In vitro* addition of GH promotes the maturation of human oocytes and may potentially increase the fertilization rate, which is in agreement with the results from other animal models, like bovine, rat, and ovine [32–35]. Similarly, in the present study, the addition of GH during *in vitro* culture improved the impeded meiosis process in aged oocytes, enabling more morphologically normal MII oocytes to develop into blastula.

However, the possibility of *in vivo* GH supplementation to improve reproductive function in women of advanced age remains controversial. In clinical treatment, GH is usually subcutaneously injected from the initiation day of ovarian stimulation to the administration of hCG, or pretreated for four to six weeks before ovarian stimulation [31]. As reported in previous studies, though the developmental potential of oocytes may be improved by GH, as indicated by the increased live birth rate, no statistical significance could be observed in the quantity of MII oocytes retrieved from elderly patients co-treated with GH [36]. Similar results were obtained in a meta-analysis by Kolibianakis et al., which showed that GH supplementation improves oocyte quality, whereas there is no increment in the number of collected cumulus–oocyte complexes (COCs) [37]. Nevertheless, in a randomized controlled trial with patients with an average age of 35 years, indicators of ovarian storage in the GH group were higher than in the normal antagonist group [38]. Overall, obvious inconsistencies can be seen between clinical outcomes, and efforts are therefore necessary to improve GH medication methods in order to achieve superior efficacy.

Though mice are a suitable model to study administration methods, dosages, and duration and to investigate ovarian storage and oocyte quality, few relevant studies have been published. The efficacy and underlying mechanisms of GH treatment in older mammals are therefore poorly understood. In the present study, the ovarian reserve of aged mice was remarkably ameliorated after eight weeks of *in vivo* GH supplementation, which was comprehensively demonstrated by the evident increases in the numbers of follicles at all stages and matured oocytes. Meiotic defects were restored, and the capacity of embryo formation was enhanced in the GH group, suggesting a vital advancement in oocyte quality. Considering the fact that *in vitro* culture experiment showed a mild improvement of oocyte development after GH treatment, while *in vivo* injection of GH increased ovarian reserve significantly, we believed that GH treatment improved not only the oocyte but also the ovarian somatic cells. More experiments should be carried to further understand the effect of GH on ovarian somatic cells, which will contribute to the clinical application. Collectively, these results are not completely consistent with the outcomes from clinical trials, implying that extended durations of GH administration may add to the improvement of ovarian functions.

Furthermore, in spite of the application of GH in clinical settings, the mechanisms by which it improves oocyte quality are not fully understood. GH can directly interact with GHRs on oocytes and granulosa cells to promote follicle proliferation through the JAK-STAT pathway, and may also have an indirect effect on oocyte quality through activating the synthesis of insulin-like growth factor (IGF-1) [39, 40]. Besides, it has been demonstrated that GH inhibits the JNK pathway through the activation of GHR in breast cancer cells [41]. Previous studies reported that the JNK pathway anticipates cell death in multiple forms, including apoptosis, necrosis, and autophagy [42]. Polypeptides involved in the JNK pathway are believed to be associated with expressions of the *Fos* and *Jun* gene families [43]. JNK/c-Jun signaling is found to be essential in promoting specific neuronal apoptosis for the development of the mammalian nervous system [44].

Activating protein-1 (AP-1), which is composed of hetero-oligomerized Fos, Jun, and ATF proteins, not only participates in cell proliferation and differentiation but also induces cell apoptosis under certain circumstances, where c-Jun and c-Fos are highly expressed [45]. The positive regulation of AP-1 in apoptosis has been shown in several biological processes. Inhibiting the expression of c-Fos and c-Jun, which suppress the activity of AP-1, promotes the survival of lymphoid cells lacking growth factors [46]. Besides, upregulation of AP-1 is associated with apoptosis of hematopoietic cells to maintain blood cell homeostasis [47].

In the present study, GH treatment prominently reduced the incidence rates of spindle abnormality and the expression of γH2AX, as well as oocyte apoptosis, as revealed by single oocyte transcriptome analysis. γH2AX is not only a marker of DNA damage but also a marker of senescence in the absence of DNA damage [48, 49]. These results indicated that GH was beneficial for aged oocytes. Further experiments indicated that the expression levels of *Fos* and *Jun* gene families were significantly downregulated under GH treatment. Our study demonstrates that GH can protect oocytes from apoptosis and counteract the effects of aging partially through the JNK pathway and its downstream effectors, namely, the members of Fos and Jun protein families. However, the specific mechanisms underlying the effects of GH on Fos and Jun require further exploration.

In conclusion, GH supplementation can ameliorate the ovarian reserve and oocyte quality in aged mice by reducing DNA damage and apoptosis. More studies should be carried to set a standard for the indications, dosages, and duration of GH treatment in clinical application.

## MATERIALS AND METHODS

### Animals

Young (6-week-old) and aged (8-month-old) C57BL/6J mice were purchased from the Experimental Animal Center of Nanjing Medical University (Nanjing, China) and Gempharmatech company (Nanjing, China) and raised in the Animal Laboratory Center of Nanjing Drum Tower Hospital (Nanjing, China) on a 12-h light:12-h dark cycle with available water and food.

### Measurement of growth hormone levels in peripheral blood

The mice were anesthetized during the diestrus at 2 p.m. Blood was collected and centrifuged at 3000 rpm for 10 min. The supernatant was collected and frozen at −80° C. After freezing on ice, the GH concentrations were measured by the mouse GH ELISA kit (Nanjing Jianchengbio, Nanjing, China).

### GH treatment

In the *in vitro* experiment, oocytes from aged (10-month-old) mice were treated with GH (Anke Biotechnology, Anhui, China) (50 ng/mL) in M2 medium. In the *in vivo* experiment, 8-month-old mice were intraperitoneally injected with GH (1 mg/kg body weight) every two days for two months. The control group was treated with NS.

### Oocyte collection and culture

Forty-eight hours after injection with 10 IU PMSG, the mice were sacrificed to obtain fully grown oocytes. GV oocytes were cultured in M2 medium (Sigma, St. Louis, MO, USA) covered by liquid paraffin oil at 37° C in a 5% CO_2_ incubator. Data were recorded for analysis at different time points (4 h for the GVBD stage, and 14 h for the metaphase II stage).

### *In vitro* fertilization and embryo culture

Mice were sacrificed by cervical dislocation, and fallopian tubes were put into 1 mL mHTF. The enlarged part of the fallopian tube was punctured with the tip of a sterile syringe, and the COC was gushed out. The COC was incubated with 80 IU/mL hyaluronidase (Sigma-Aldrich) for about 2–3 minutes, and then the oocytes were transferred to mHTF medium with a fine glass needle and washed three times to remove granulosa cells. All MII oocytes were collected for further research. Male C57BL/6J mice (3–4 months old) were used to acquire sperm. Sperm cells were cultured for 1 h to achieve capacitation, and then adjusted to a concentration of about 1×10^6^/mL. To each droplet of COC, 6 μL of sperm cell suspension was added. The fertilized oocytes were transferred into HTF for 5 h at 37° C in a 5% CO_2_ incubator. Then the two-cell embryos were cultured in KSOM medium (Sigma). The fertilization rate, cleavage rate, and blastocyst formation rate were calculated.

### Estrous cycle analysis, body weight, and ovarian index

Vaginal smears were applied to examine the estrous cycle for eight days. The mice were anesthetized and weighed at diestrus, and a U-shaped incision in the lower abdomen was made to expose the general organs of the abdominal cavity. The left ovary was completely taken out and fixed in 4% formaldehyde solution. The right ovary was put into liquid nitrogen and stored at −80° C. The following formula was used to calculate the ovarian index: ovarian index = ovarian wet weight (mg)/body weight (g) × 100%.

### Histological analysis of ovaries and follicle counting

Ovaries were cut into 5 μm slices, and one of every five sections was used as a histologic slice. Ovarian slides were deparaffinized in xylene and ethanol in sequence, and then stained with H&E. Tissue slides were observed under a light microscope with a magnification of 200×, and every follicle was classified by stage. Follicle counting was performed using an unbiased stereological method, and the average of five times counting was taken as the whole-ovary follicle counting results.

### Immunohistochemistry

Slides were deparaffinized in xylene and ethanol in sequence, and 3% hydrogen peroxide was used to block endogenous peroxidase for 10 min. Samples were autoclaved with citrate buffer solution (pH 6.0) for antigen retrieval and blocked with goat serum for 30 min. Tissue slides were immunostained with primary antibodies overnight at 4° C. The following primary antibodies and dilutions were used: rabbit anti-Ki67 (1:200; Abcam, Cambridge, UK), rabbit anti-c-Fos (1:200; Abways, Shanghai, China), rabbit anti-c-Jun (1:200; Abways), rabbit anti-Fosb (1:100; Santa Cruz, Dallas, TX, USA), and mouse anti-Junb (1:100; Santa Cruz). The slides were washed with PBST. Goat anti-rabbit/mouse IgG was applied to the slides for 30 min at room temperature. Diaminobenzidine was used to detect HRP activity and hematoxylin to counterstain the slides. Digital images were captured using a Leica DM 2000 microscope (Lecia, Wetzlar, Germany). The measurement of protein expression was performed using ImageJ software (NIH, Bethesda, MD, USA) in a blind fashion.

### Immunofluorescence staining

Ovarian slides were stained with mouse anti-γ-H2AX (1:200; Millipore, Temecula, CA, USA) at 4° C overnight. On the following day, slides were incubated with Alexa Fluor 594-conjugated goat anti-mouse IgG (Life Technology, Waltham, MA, USA) at room temperature in the dark. Hoechst was used to stain the nuclei. Digital images were captured using a Leica DM 3000 LED microscope (Lecia).

### Oocyte immunofluorescence

Oocytes were fixed in 4% paraformaldehyde in PBS for 30 min, followed by permeabilization in 0.5% Triton X-100 for 20 min. After washing with 1% BSA for three times (3–5 min each) and blocking with 1% BSA for 1 h, oocytes were treated with anti-α-tubulin (1:200; Sigma) or mouse anti-γ-H2AX (1:200; Millipore) at 4° C overnight. After washing with PBST for three times (3–5 min each), oocytes were incubated with secondary antibody (1:200) diluted in PBST for 1 h at room temperature. Oocytes were washed with PBST for another three times and counterstained with PI or Hoechst for 10 min. At last, oocytes were mounted on glass slides and observed under a Leica DM 3000 LED microscope (Lecia).

### Single oocyte quantitative real-time PCR

All primers were mixed with nuclease-free water to a final concentration of 0.1 μM. cDNA was extracted from single oocytes using a single cell sequence-specific amplification kit (Vazyme, Nanjing, China), and qPCR was performed on a detection machine (Analytik Jena, Jena, Germany). The following primer sequences were used: *c-Fos*: forward, 5′-CTGAAGCTGACTCCTTCCCA-3′, reverse, 5′-TTGCCTTCTCTGACTGCTCA-3′; *c-Jun*: forward, 5′-CGCTGGAAAGCAGACACTTT-3′, reverse, 5′-TGGGTCCCTGCTTTGAGAAT-3′; *Fosb*: forward, 5′-CAGGAGTTGGGATGGAGGAG-3′, reverse, 5′-AACCACTCCTGGCTTATGCT-3′; *Junb*: forward, 5′-TCTACACCAACCTCAGCAGT-3′, reverse, 5′-ATGTGGGAGGTAGCTGATGG-3′; *Jund*: forward, 5′-TCCCAACTCTCCTCTGACCT-3′, reverse, 5′-TGCTGGTGTGTTTGTCTGTG-3′; *Lmnb2*: forward, 5′-CGTGACAAGTTCCGCAAGAT-3′, reverse, 5′-ATGTCCAGGGCCAGCTTAAT-3′; *18S*: forward, 5′-ATGGCCGTTCTTAGTTGGTG-3′, reverse, 5′-CGGACATCTAAGGGCATCAC-3′. The relative expression levels were calculated via the 2^−ΔΔCt^ method.

### Single-cell RNA sequencing of transcriptome libraries and analysis

We used the Discover-sc^TM^ WTA Kit V2 (Vazyme, N711) to obtain cDNA from single MII oocytes. Then the TruePrep^TM^ DNA Library Prep Kit V2 for Illumina (Vazyme, TD503) was used to make libraries. The libraries were sequenced using an Illumina HiSeq X platform (Shanghai, China). We aligned high-quality reads to *Mus musculus* UCSC mm9 references and calculated the FPKM value of each gene. All of the subsequent analyses were based on high-quality sequences.

### Statistical analysis

We calculated the mean and standard deviation of the experimental variables. Statistical analyses were performed by Student’s *t* test using GraphPad Prism 8 statistical software (San Diego, CA, USA). *P* < 0.05 was considered to indicate statistical significance.

## Supplementary Material

Supplementary Figure 1

Supplementary Table 1
