# Supplementary material for: Growth hormone ameliorates the age-associated depletion of ovarian reserve and decline of oocyte quality via inhibiting the activation of Fos and Jun signaling
Source: Aging (Albany NY). 2021 Feb 17;13(5):6765–81. doi: 10.18632/aging.202534 (PMC7993724; doi:10.18632/aging.202534)
Supplement: Supplementary Figure 1 [file aging-13-202534-s001.pdf]

SUPPLEMENTARY FIGURE

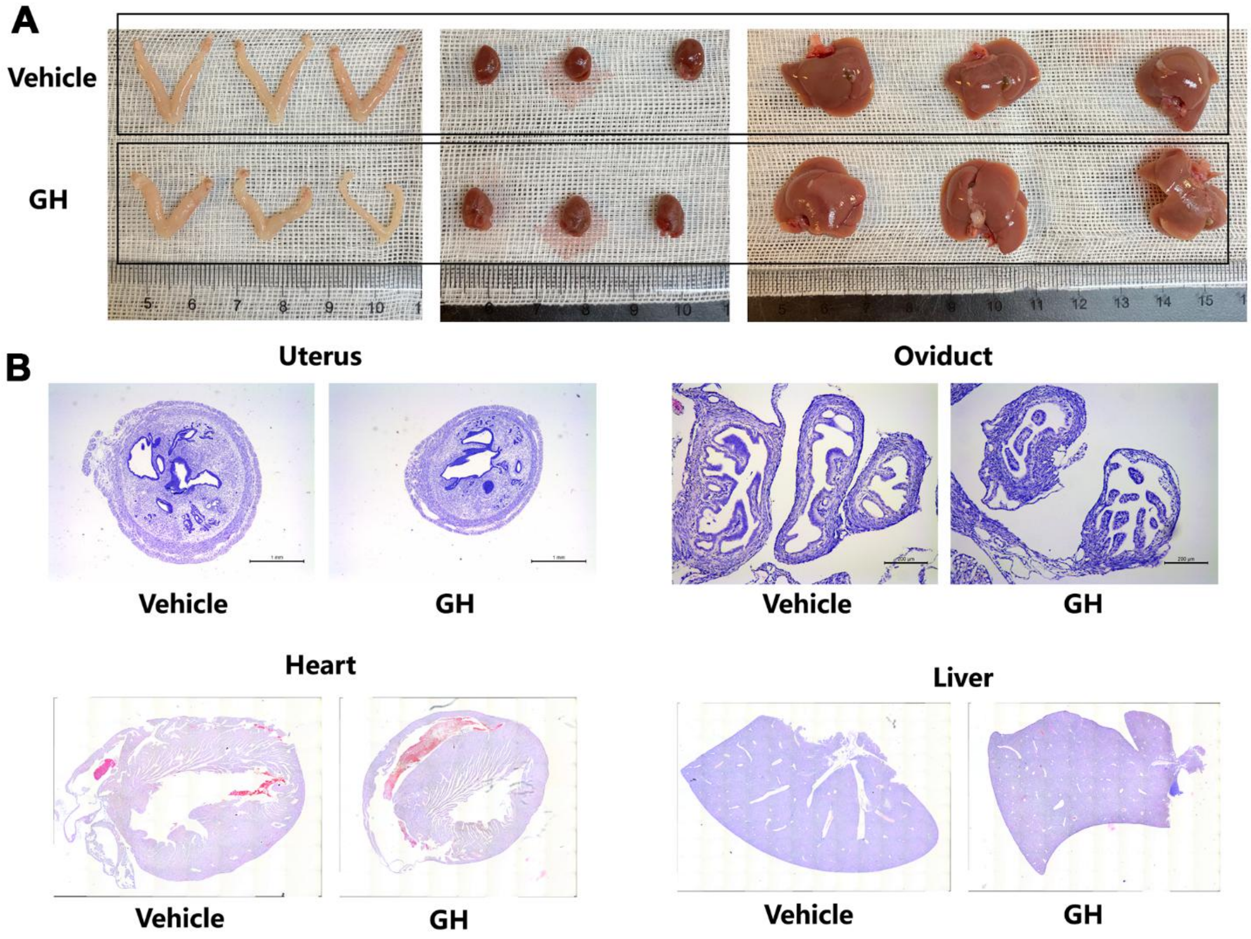

Supplementary Figure 1. Effect of GH treatment on other organs. (**A**) Micrographs of NS-treated and GH-treated mouse uterus, heart, and liver. (**B**) HE-stained of NS-treated and GH-treated mouse uterus, oviduct, heart, and liver (Scale bar, 1 mm, 200 µm).
